# The effect of training for a participatory ergonomic intervention on physical exertion and musculoskeletal pain among childcare workers (the TOY project) – a wait-list cluster-randomized controlled trial

**DOI:** 10.5271/sjweh.3884

**Published:** 2020-07-01

**Authors:** Charlotte Diana Nørregaard Rasmussen, Ole Henning Sørensen, Allard J van der Beek, Andreas Holtermann

**Affiliations:** National Research Centre for the Working Environment, Lersø Parkallé 105, 2100 Copenhagen Ø, Denmark; Amsterdam UMC, Vrije Universiteit Amsterdam, Department of Public and Occupational Health, Amsterdam Public Health Research Institute, van der Boechorststraat 7, 1081 BT, Amsterdam, The Netherlands

**Keywords:** Key terms MSD, musculoskeletal disease, musculoskeletal disorder, RCT, sickness absence, workplace intervention

## Abstract

**Objective:**

Many employees have high physical exertion at work and suffer from musculoskeletal pain (MSP) leading to sickness absence with large costs. Participatory ergonomics is a potentially effective intervention for reducing physical exertion, MSP and sickness absence. The main aim of this study was to investigate the effectiveness of a 20-week workplace participatory ergonomic intervention among childcare workers on physical exertion and MSP.

**Methods:**

In a two-arm cluster-randomized trial, 190 workers were recruited from 16 childcare institutions and randomly assigned to either a 20-week participatory ergonomics intervention consisting of three training workshops or a control group receiving usual care. Primary outcomes were physical exertion during work, maximal pain intensity, number of pain regions, and pain-related work interference. Secondary outcomes were MSP-related sickness absence, need for recovery (NFR), employee involvement, and self-efficacy. We followed the intention-to-treat principle and adhered to the registered study protocol (ISRCTN10928313).

**Results:**

After 20 weeks, half the workers noticed some positive changes in their work. However, there were no statistically discernible effects in physical exertion, maximum pain intensity, pain-related work interference, or number of pain regions. We found a significant reduction of MSP-related sickness absence in the intervention compared to the control group [-0.48 days per month (95% confidence interval (CI), -0.8– -0.1]. We found no significant effects in NRF or involvement of employees, but self-efficacy was reduced in the intervention compared to the control group [-0.2 (95% CI, -0.3– -0.0)].

**Conclusion:**

This 20-week training for a participatory ergonomic intervention in childcare workers did not show effects on physical exertion and MSP, but was both feasible and effective in reducing MSP-related sickness absence.

There are more than 671 million children under five years of age in the world today. Given that labor force participation rates exceed 60% globally, a large number of these children need some sort of non-parental care during the day (1). In particular womens’ labor force participation is highly depended on availability of high quality childcare (2). Thus, there is a great need for healthy and fit childcare workers. However, childcare workers generally appear to suffer from poor health (3, 4). Danish childcare workers report a high prevalence of musculoskeletal pain (MSP) and sickness absence (5). Preventive initiatives to improve health are, therefore, important for this occupational group (6). The workplace holds great potential for addressing these health issues and promoting longer working lives.

MSP is a main contributor to sickness absence (7), and work-related factors are among the most important intervention targets to prevent MSP and MSP-related sickness absence (8). These factors particularly involve high physical workload [eg, physical exertion (8, 9)], which has been found to be prevalent among childcare workers (5, 10, 11). The physical demands of working in childcare include the need to lift, carry, and support children in a range of activities, requiring several demanding body postures and movements, such as bending forward and twisting of the back and sitting on the floor (10). However, childcare work can also be mentally and emotionally exhausting and stressful for some individuals (12). Another important factor in those exposed to high physical work demands is the need for recovery (NFR) after a workday, which is greatest among workers who experience high levels of time pressure and physical work demands (13). Moreover, high NFR after work is associated with MSP (14) and increases the risk of subsequent sickness absence (15). Thus, there is a need for effective and feasible interventions to reduce high physical exertion during work and NFR after work, thereby preventing MSP and reducing sickness absence due to MSP among childcare workers.

Participatory ergonomics programs are commonly used as workplace interventions for prevention of MSP (16, 17). The involvement of workers in the process is essential as it ensures that participants take responsibility for and ownership of risk identification, solution development, and implementation of change (18), all of which is important for intervention effectiveness (19, 20). The participatory ergonomics process encourages workers to be involved in optimizing their own work routines, consequently decreasing work-related risk factors (21) and thereby improving their health (22). However, evidence on the effectiveness of participatory ergonomics for reducing physical exertion, MSP, NFR and MSP-related sickness absence is incomplete (16, 23–25).

Our aim of study was to investigate the effectiveness of a participatory ergonomic intervention at the workplace over 20 weeks in childcare workers on the primary outcomes physical exertion and MSP and the secondary outcomes MSP-related sickness absence, NFR, employee involvement, and self-efficacy. We hypothesized that the implementation of the 20-week participatory ergonomic intervention would reduce physical exertion and MSP among childcare workers compared to usual practice (26).

## Methods

Between August 2017 and July 2018, we conducted a two-arm, cluster-randomized controlled study with a waiting-list control. Clusters were formed based on childcare institutions and randomly assigned to two different arms (immediate versus delayed intervention, 20 weeks apart). This design yielded the possibility to offer the intervention to the control group after the intervention had been implemented in the intervention group, thereby decreasing the risk of hampering implementation due to logistical issues and reduced organizational commitment (27, 28). We published a study protocol prior to enrolling participants (26). The trial was prospectively registered (ISRCTN10928313). The Danish National Committee on Biomedical Research Ethics (ie, the local ethics committee of Frederiksberg and Copenhagen) has evaluated a description of the study and concluded that, according to Danish law as defined in Committee Act ¦ 2 and ¦ 1, the intervention described need not be reported to the local ethics committee (Ref number: 16048606). We obtained written, informed consent from all participants before they enrolled in the trial.

### Participants

Details regarding the recruitment procedures of workplaces (childcare institutions) and workers have been reported elsewhere (26). In short, the childcare institutions were recruited with assistance from the municipality of Copenhagen after presenting the project at a meeting of region managers. Eligibility criteria for participation in the study for the institutions were: (i) childcare for children aged 0–3 years, (ii) ≥9 employees (childcare workers), and (iii) no recent (within the previous year) participation in an ergonomics course from the Work Environment Consultancy of Copenhagen. There were 29 eligible institutions in total, and all their childcare workers were eligible for participation. Since this was an organizational intervention, all childcare workers were expected to participate. Due to the design of the intervention, we only included those workers who we suspected would be at the workplace during the intervention period, meaning that if we knew that a worker would end her/his employment during the study period, he/she was not included in our study sample for the trial (ie, the evaluation) but could still participate in the activities at work.

### Randomization and blinding

For practical reasons, the baseline measurement took place after randomization but before any intervention treatment. This was done because the workplaces needed to plan the workshops that were carried out as part of the intervention in advance. All childcare institutions (clusters) gave initial agreement to participate before we performed the randomization. Since the intervention was group-based, and to avoid contamination between workers, the randomization was performed across clusters at the childcare institution level balanced on size. The study was dimensioned to enroll approximately 200 workers. An independent data manager performed the randomization by using a computer-generated randomization using the SAS statistical software for Windows 9.4 (SAS Institute, Cary, NC, USA) developed by an independent statistician. Blinding of participants was not possible due to the nature of the intervention. However, data collection was performed using text messages and all persons collecting/handling data were blinded to group allocation.

### Procedures

All childcare workers in the intervention group were involved in the participatory ergonomics process. Ergonomic consultants from the Work Environment Consultancy of Copenhagen (occupational therapists and physiotherapists) guided the process. The participatory ergonomic process followed 6 steps: (i) identification of risk factors, (ii) analysis of risk factors, (iii) solution building, (iv) prototype implementation, (v) prototype evaluation, and (vi) solution adoption. A main feature of this participatory ergonomics intervention was the integration with the core work tasks as previously recommended for improving implementation (6, 29). The first workshop lasted 3 hours and was conducted in week 2. The two follow-up meetings lasted 1.5 hours each, the first meeting was conducted approximately six weeks after the first workshop and the final meeting was conducted approximately four weeks after the second meeting. In addition, each workplace was offered one ergonomic consultant visit. We observed a selection of workshops and assessed the content against pre-specified criteria to check the workshop content was as intended (fidelity). Those in the control group followed usual practice from baseline to 20-week follow-up. This group received the intervention after the 20-week follow-up. More information about the intervention can be found in the study protocol (26).

### Outcomes and measurements

Data were collected at 4, 8, 12, 16, and 20 weeks after randomization by use of electronic questionnaires sent via text message to participants’ mobile phone (a link to a questionnaire in survey exact). The two primary outcomes of this study were self-rated physical exertion measured on a 0–10 Likert scale (30) and MSP, measured as: (i) maximal pain intensity [0–10 on a numeric rating scale (NRS)] in any one of eight body regions (low back, neck, shoulders, knees, elbows, hands, hips, feet/ankles), (ii) number of pain regions (calculated as number of pain regions with an episode of pain (defined as >1 day with pain in a body region and with an intensity of ≥3 on the NRS), and (iii) pain-related work interference (days in the previous four weeks with pain that limits ability to do the work). Secondary outcomes were: (i) self-reported sickness absence due to MSP (days) (31) measured by questionnaire every four weeks from baseline to week 20; (ii) self-efficacy (32) measured by questionnaire at baseline and at week 20; (iii) NFR (33, 34) measured by questionnaire at baseline and at week 20; and (iv) employee involvement (35) measured by questionnaire at baseline and at week 20.

### Process measures – changes in work

At week 20, the intervention group was asked to score statements about changes in work adapted from Nielsen & Randall’s framework (36) about implementation, which was developed specifically for organizational level occupational health interventions. The statements posed were: (i) Through the implementation of the intervention, we finally get to straighten up some bad work methods that we had accepted; and (ii) New procedures have been introduced after the implementation of the intervention. The answer categories were on a 5-point Likert-type scale, from strongly disagree to strongly agree.

### Statistical analyses

A sample of 192 participants (96 per group) corresponding to approximately 16 clusters in total was required to ensure 80% power to statistically demonstrate a relevant effect in physical exertion of 1 point (20). We estimated the effect of the intervention on the primary outcome using a mixed model for repeated measures. We treated time as a categorical variable (week 4, 8, 12, 16, and 20) and included group × time interactions to determine treatment effects at each time point. In addition, we took into account the possible differences between the groups at baseline. For this, a model was developed in which the treatment variable was not part of the model, but its interaction with time was (37). We set statistical significance at P<0.05 for a 2-sided test. The primary analysis was by intention-to-treat, including all eligible randomized participants who provided follow-up data. We compared demographic characteristics between dropouts and completers.

## Results

**Figure 1:**
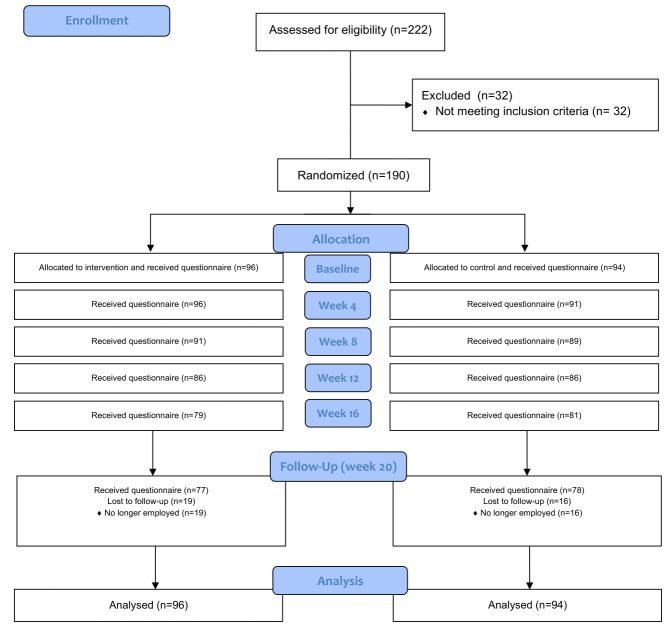
Consort flow diagram.

Figure 1 shows the flowchart of the trial. Of 222 eligible workers from 16 workplaces, 190 (86%) wanted to participate and provided baseline data; 32 were excluded for lack of data. In total, 96 and 94 childcare workers were randomized to the intervention and control groups, respectively. After the 20-week intervention period, there were 19 (20%) and 16 (17%) workers lost to follow-up in the intervention and control groups, respectively. Table 1 shows baseline characteristics of the workers in both groups. There was a slight difference between the two groups with respect to gender. However, other demographic variables were similar. For both the primary and secondary outcomes there were some differences between the groups at baseline. We controlled for this difference in the statistical analysis by including baseline data of the respective variables in the model.

**Table 1:** Baseline characteristics.[SD=standard deviation.]

	Intervention group (N=96)		Control group (N=94)	
	
Mean (SD)	N (%)	Mean (SD)	N (%)
Age (years)	37 (12.2)		38 (11.5)	
Gender (female)		78 (81)		88 (94)
Ethnicity (born in Denmark)		87 (91)		77 (82)
Smokers		26 (27)		19 (20)
Body mass index (kg/m^2^)	25.7 (5.2)		25.0 (5.9)	

There were small differences in baseline characteristics between dropouts and completers. Dropouts were younger than the completers (28 versus 39 years). On other baseline characteristics the two groups were similar (data not shown).

### Process measures

*Dose delivered and dose received*. The intervention was delivered by three ergonomic consultants from the Work Environment Consultancy of Copenhagen. All planned activities were delivered (table 2). In total, 88%, 70% and 62% of the workers participated in the first, second and third workshops, respectively. In addition, 43 (45%) of the workers participated in all three workshops, 32 (33%) participated in two workshops, 17 (18%) participated in only one workshop, while four (4%) did not participate in any of the workshops (data not shown). The reasons for not participating in the workshops were employment ceased, vacation, leave, sickness absence or other/unknown.

**Table 2:** Participants in workshop.

Intervention activities	Dose delivered		Dose received (N=96)
	
	Planned	Delivered (%)	N (%)
Workshop 1	8	100	84 (88)
Workshop 2	8	100	67 (70)
Workshop 3	8	100	59 (62)

*Appraisal of intervention*. Most participants were satisfied with the intervention (78%) and found it relevant (82%). In addition, nearly all (92%) of the participants considered the intervention to be relevant for other childcare institutions (data not shown).

*Changes in work*. After implementation of the intervention, 58% of the participants agreed they had finally addressed some bad work methods they had previously accepted, and 50% agreed that new procedures had been introduced.

*Intervention effects*. Table 3 shows the intervention effects. At week 20, there were no statistically significant effects in physical exertion, maximum pain intensity, pain-related work interference, or number of pain regions. However, the estimates in mean treatment effect between groups at week 20 showed a small reduction in physical exertion and maximum pain intensity in the intervention group.

**Table 3:** Intervention effects on physical exertion, pain-related work interference, number of pain regions, maximum pain intensity, and sickness absence due to musculoskeletal pain (MSP), self-efficacy, need for recovery and employee involvement. [SE=standard error; CI=confidence interval]

Variable	Time	Intervention group		Control group		Mean treatment difference between group		
		
		N	Estimate (SE)	N	Estimate (SE)	Estimate (SE)	95% CI	P-value
Primary outcomes								
Physical exertion (0–10)	Baseline	96	5.6 (1.8)	94	6.2 (1.6)			
	Week 20	61	5.5 (2.0)	61	6.1 (1.9)	-0.2 (0.3)	-0.8−0.4	0.45
Pain-related work interference (days 0–28)	Baseline	95	2.3 (5.2)	94	4.7 (7.70)			
	Week 20	61	3.7 (6.9)	60	4.6 (7.55)	0.2 (1.2)	-2.1−2.5	0.59
Number of pain regions (0–8)	Baseline	95	2.2 (1.7)	94	2.6 (1.83)			
	Week 20	61	2.2 (2.0)	60	2.5 (2.09)	0.1 (0.2)	-0.4−0.5	0.84
Maximum pain intensity (0–10)	Baseline	95	5.4 (2.30)	94	5.9 (2.8)			
	Week 20	61	5.2 (2.7)	60	5.6 (3.2)	-0.1 (0.4)	-0.9−0.7	0.73
Secondary outcomes								
Sickness absence due to MSP (days 0–28)	Baseline	94	0.4 (2.3)	94	0.7 (3.2)			
	Week 20	61	0.1 (0.4)	60	0.4 (1.1)	-0.4 (0.2)	-0.6− -0.1	0.01
Self-efficacy (0–4)	Baseline	96	3.3 (0.5)	94	3.4 (0.5)			
	Week 20	62	3.3 (0.6)	67	3.4 (0.5)	-0.2 (0.1)	-0.3− -0.0	0.01
Need for recovery (0-100)	Baseline	96	60.94 (29.20	94	63.12 (26.98)			
	Week 20	62	62.37 (31.21)	67	64.43 (25.28)	1.30 (2.41)	-3.45−6.04	0.59
Employee involvement (0–100)	Baseline	96	73.09 (17.33)	94	66.49 (16.35)			
	Week 20	62	74.29 (15.02)	67	70.06 (14.66)	1.94 (1.91)	-1.81−5.69	0.31

We found statistically significant effects in the intervention group on reduction of sickness absence due to MSP at week 20 compared to the control group. This corresponded to an intervention effect after 20 weeks for sickness absence due to MSP of -0.4 days per month [95% confidence interval (CI), -0.8– -0.1].

At week 20, there were no significant effects in NFR or involvement of employees. However, there was a statistically significant reduction in self-efficacy of -0.2 (95% CI, -0.3– -0.0) in the intervention compared to control group.

## Discussion

We hypothesized that the implementation of the 20-week participatory ergonomic intervention would reduce physical exertion and MSP among childcare workers compared to usual practice. This hypothesis was not confirmed. Nevertheless, the participatory ergonomic intervention was both feasible and effective in reducing MSP-related sickness absence.

Our findings contrast with a systematic review concluding that participatory ergonomics was effective in reducing MSP (16). However, the results obtained from our study are in accordance with other randomized controlled trials, reporting that participatory ergonomics was not effective in reducing MSP (24, 38).

By introducing participatory ergonomics, we aimed to minimize risk factors for MSP at work and improve the work tasks perceived as physically demanding. After the intervention, no significant effect was found for physical exertion or MSP when comparing the intervention with the control groups. In addition to these findings, we did not find any significant effects on NFR. According to our program logic, due to the participatory nature of the intervention, we expected the intervention to have an effect on self-efficacy and employee involvement (26). However, this was not the case. On the contrary, we found a small reduction of 0.15 on a scale from 0–4 in self-efficacy in the intervention group. It is hard to interpret if this change is an intervention effect or just a chance finding. Furthermore, we question whether such a small reduction is of any practical relevance.

When an intervention does not show an effect on the primary outcomes, it is important to consider whether this is a theory or implementation failure (29, 39, 40). With respect to implementation, all intervention workshops were successfully delivered. However, the dose received decreased over time, resulting in only 62% of the participants participating in the last workshop. This number, however, is comparable to other participatory ergonomics interventions (41). In addition, the measures regarding changes in work showed that 50–58% of the participants said the intervention resulted in changes in their work. This number might point towards implementation failure, since only half of the childcare workers said that changes had been made. In general, many ergonomic intervention studies lack information about implementation (17, 42). In our study, we have also gathered information about specific work exposures by objective measurements. However, there is a need for a deeper investigation of specific exposures related to the intervention. This is out of the scope of this paper but will be reported in a separate paper (26).

With respect to theory failure, one reason may be related to difficulties in evaluating participatory interventions. Due to the participatory approach, we do not know much about the actual content of a participatory ergonomic intervention, eg, risk identification or solution development, which then becomes a black box (43). Therefore it is important to consider whether the chosen outcomes are the most optimal for evaluating the effectiveness of participatory ergonomics interventions.

The participatory ergonomic intervention was effective in reducing MSP-related sickness absence. This finding is in accordance with another study among employees in pre-schools from Denmark investigating a participatory organizational level intervention with a focus on the core task at work (6). A main feature of our participatory ergonomics intervention was also the integration with the core work tasks. This was, in particular, how to make the children more independent of active assistance from the childcare workers (eg, climbing up in the crib and changing table, getting outdoor clothes on) so that the physical work demands could be decreased among the childcare workers. The intervention effect on MSP-related sickness absence could therefore be explained by the children requiring less assistance, possibly making the childcare worker better able to work with the same level of MSP. The reduction in MSP-related sickness absence after 20 weeks was very high (corresponded to a reduction of 88% from baseline to follow-up) and thus of substantial importance for the workplace. However, we do not know whether it was the focus on the core tasks at work that resulted in the reduced MSP-related sickness absence or if other mechanisms were at work. This should be investigated by further analyses of the process evaluation data from the study.

Another main finding of the study was the high feasibility of implementing the participatory ergonomic intervention in terms of high delivery of the intervention and the moderate dose received. This is possibly also related to the focus on the core work tasks, making the intervention more relevant for the childcare workers, and not considering sideline activities with limited relevance (29). This was also seen in the positive appraisal of the intervention with nearly all (92%) of the participants considering the intervention to be relevant for other childcare institutions

### Strengths and limitations of the study

The cluster-randomized controlled trial design is a methodological strength since it minimized the risk of contamination between the intervention and reference group and reduced the risk for bias. Repeated measurements with short recall were used to measure study outcomes and we used measures that have all been found to have a reliable validity (30, 31). Another strength is that consultants delivered the intervention and were not involved in the evaluation. Lastly, this study was executed in real working-life settings, which makes it easier to generalize the effects to similar workplaces.

A limitation of this study is the loss to follow-up rates on the primary and secondary outcomes found after 20 weeks. Unfortunately, loss to follow-up is a common problem among prevention studies (44). Checking our data for selective dropout revealed that dropouts did not differ from completers. Also, since this is an organizational intervention, it did not focus on individual workers. Thus, individual randomization was not feasible. Moreover, due to the interventional trial design, participants were not blinded to group allocation. Finally a limitation is that we did not measure changes in work very well.

### Concluding remarks

A 20-week workplace participatory ergonomic intervention in childcare workers did not show effects on the primary outcomes of physical exertion and MSP, but was both feasible and effective in reducing MSP-related sickness absence.
